# A retrospective case study of successful translational research: Gazelle Hb variant point-of-care diagnostic device for sickle cell disease

**DOI:** 10.1017/cts.2021.871

**Published:** 2021-10-25

**Authors:** Kelli Qua, Shannon M. Swiatkowski, Umut A. Gurkan, Clara M. Pelfrey

**Affiliations:** 1 Clinical and Translational Science Collaborative, School of Medicine, Case Western Reserve University, Cleveland, OH, USA; 2 Department of Mechanical and Aerospace Engineering, School of Engineering, Case Western Reserve University, Cleveland, OH, USA; 3 Department of Biomedical Engineering, Case School of Engineering, Case Wetern Reserve University, Cleveland, OH, USA; 4 Case Comprehensive Cancer Center, Case Western Reserve University, Cleveland, OH, USA

**Keywords:** Retrospective case studies, sickle cell disease, successful translation, CTSA, pilot program, point-of-care diagnostics

## Abstract

Evaluation researchers at Clinical and Translational Science Award (CTSA) hubs are conducting retrospective case studies to evaluate the translational research process. The objective of this study was to deepen knowledge of the translational process and identify contributors to successful translation. We investigated the successful translation of the HemeChip, a low-cost point-of-care diagnostic device for sickle cell disease, using a protocol for retrospective translational science case studies of health interventions developed by evaluators at the National Health Institutes (NIH) and CTSA hubs. Development of the HemeChip began in 2013 and evidence of device use and impact on public health is growing. Data collection methods included five interviews and a review of press, publications, patents, and grants. Barriers to translation included proving novelty, manufacturing costs, fundraising, and academic-industry relations. Facilitators to translation were CTSA pilot program funding, university resources, entrepreneurship training, due diligence, and collaborations. The barriers to translation, how they were overcome, and the key facilitators identified in this case study pinpoint areas for consideration in future funding mechanisms and the infrastructure required to enable successful translation.

## Introduction

Case studies are a valuable method for investigating contributing factors in the research process. Case studies of translational science are used to examine elements that advance science and improve public health outcomes. Recently, a standardized protocol for conducting retrospective case studies to evaluate the translational research processes was published [1]. Following this protocol, we conducted a retrospective case study of the HemeChip, a low-cost point-of-care (POC) diagnostic test for sickle cell disease (SCD). Data collection methods included interviews, publicly available data, and document reviews with a focus on information from initial conceptual work (2013) to present day. Results are presented as a timeline of major events in the HemeChip’s translation, key scholarly products of the research team, facilitators and barriers to translation, and evidence of the current state of dissemination and implementation. The objective of this study was to deepen knowledge of the translational process and identify contributors to successful translation.

### Sickle Cell Disease

Hemoglobin (Hb) disorders are among the most common genetic diseases. Nearly 7% of the people in the world carry hemoglobin gene variants. Most prevalent hemoglobin variants are the recessive β-globin gene mutations, βS or S, βC or C, and βE or E [2,3]. Genetic disorders, such as SCD, are among the major causes of anemia [4–7].

Sickle hemoglobin variant (Hemoglobin S) is prevalent in sub-Saharan Africa [8] and in tribal populations of Central India [9]. Hemoglobin C variant is common in West Africa [10], and Hemoglobin E is common in Southeast Asia [11]. Hemoglobin S results from a point mutation in the 6th codon on the β-globin gene replacing the normal amino acid glutamine with valine amino acid, which is hydrophobic [12]. SCD emerges when such mutations are inherited from both parents, homozygously (Hb SS) or together with another β-globin gene mutation, such as hemoglobin C (Hb SC) or β-thalassemia (compound heterozygous, Hb Sβthal+/0). In SCD, abnormal polymerization of deoxygenated sickle hemoglobin results in abnormal red blood cells (RBCs), which alters the RBC shape, and triggers inflammation and endothelial cell activation [13]. These RBCs are stiff and adhesive in the small blood vessels, particularly where the oxygen tension is relatively low, such as the kidney or spleen [13]. These abnormalities result in vascular occlusion and can stop blood flow to vital organs [14]. In childhood, vascular occlusions in the spleen increase the risk for life-threatening infections [15,16]. Individuals who survive to adulthood suffer from painful crises, cumulative organ damage, and early mortality [17,18]. These complications can be mitigated by early diagnosis and comprehensive medical care [18–20]. Individuals who inherit one copy of hemoglobin S and one copy of the normal Hemoglobin A have sickle cell trait (Hb AS or SCD Trait). These people are healthy carriers but have a 25% chance of transmitting SCD to their offspring.

In the USA, most cases of SCD are reported in African Americans, but the condition is also common in Hispanics [21]. SCD affects about 100,000 Americans, and one of every 365 African Americans and one of every 16,300 Hispanic births have SCD [21]. One in 13 African Americans carry the sickle cell genetic trait. Over 300,000 babies with severe hemoglobin disorders are born each year [22,23]. SCD is a lifelong condition for millions of people worldwide and requires early detection and treatment to improve overall well-being.

### SCD Diagnosis and Treatment

Geographically, countries with some of the lowest gross domestic product (GDP) report the highest prevalence rates of SCD. These countries are unable to implement costly, centralized SCD screening programs [24]. The World Health Organization (WHO) designated SCD as a global public health problem in 2006 when it was reported that 70% of early SCD-related mortalities could have been prevented by implementing low-cost screening followed by cost-effective treatments [25]. Traditionally, SCD has been diagnosed using one of the following methods: hemoglobin electrophoresis, high-performance liquid chromatography (HPLC), or isoelectric focusing and molecular approaches. In 2019, the WHO listed hemoglobin electrophoresis as an essential in vitro diagnostic test for SCD and sickle cell trait in low- and middle-income countries [26]. Each of these methods is cost- and time-intensive, requiring not only experienced staff but also extensive resources [27].

SCD causes the highest morbidity and mortality among hemoglobin disorders [28]. While some medications mitigate the symptoms of SCD, the only cure for SCD is a bone marrow transplant. Recently, genetic and cellular therapies, including CRISPR-Cas9-based curative treatments, are being tested in both USA and European clinical trials. An estimated 50–90% of these babies die before age 5, in part because they are not diagnosed and hence not treated [25,29–32]. It is projected that by 2050, about 400,000 babies will be born with SCD annually worldwide [18,33].

### HemeChip: A Novel SCD Diagnostic Tool

The HemeChip is a reliable and affordable diagnostic tool designed for POC use [6]. The HemeChip uses electrophoresis testing and is a compact, user-friendly, and low-cost mass-producible platform. The HemeChip identifies SCD and other critical hemoglobinopathies, and it also provides relative percentages of hemoglobin types. The compact single-use microchip within the HemeChip platform contains cellulose acetate paper, in which the hemoglobin separation takes place, as well as integrated stainless-steel electrodes. The HemeChip test is performed in four steps: a finger prick equivalent amount of whole blood (diluted and lysed) is applied to the microchip; the microchip is placed inside the HemeChip reader; real-time images of the hemoglobin electrophoresis are captured during the test by the portable reader; and custom built-in software automatically analyses the results, extracts relevant peak positions, and performs quantitative analysis similar to HPLC, which is the clinical gold standard. The HemeChip reports rapid screening results at the POC in less than ten minutes, for the cost of about a few dollars per test, and can be administered by minimally trained personnel [34].

The HemeChip name is an abbreviation of and represents miniaturized hemoglobin electrophoresis in a microchip format. This name was used by the Case Western Reserve University (CWRU) research team during initial research and development phase. The HemeChip has been commercialized under the product name “Gazelle Hb Variant” by Hemex Health (Portland, Oregon), after extensive market and branding research carried out in India and Africa. The HemeChip name was retired after “Gazelle Hb Variant” was adapted by Hemex Health, the eventual licensing company. To date, there is no other POC diagnostic technology that can provide quantitative, accurate, affordable screening of hemoglobin variants.

## Case Study Findings

**Fig. 1.**
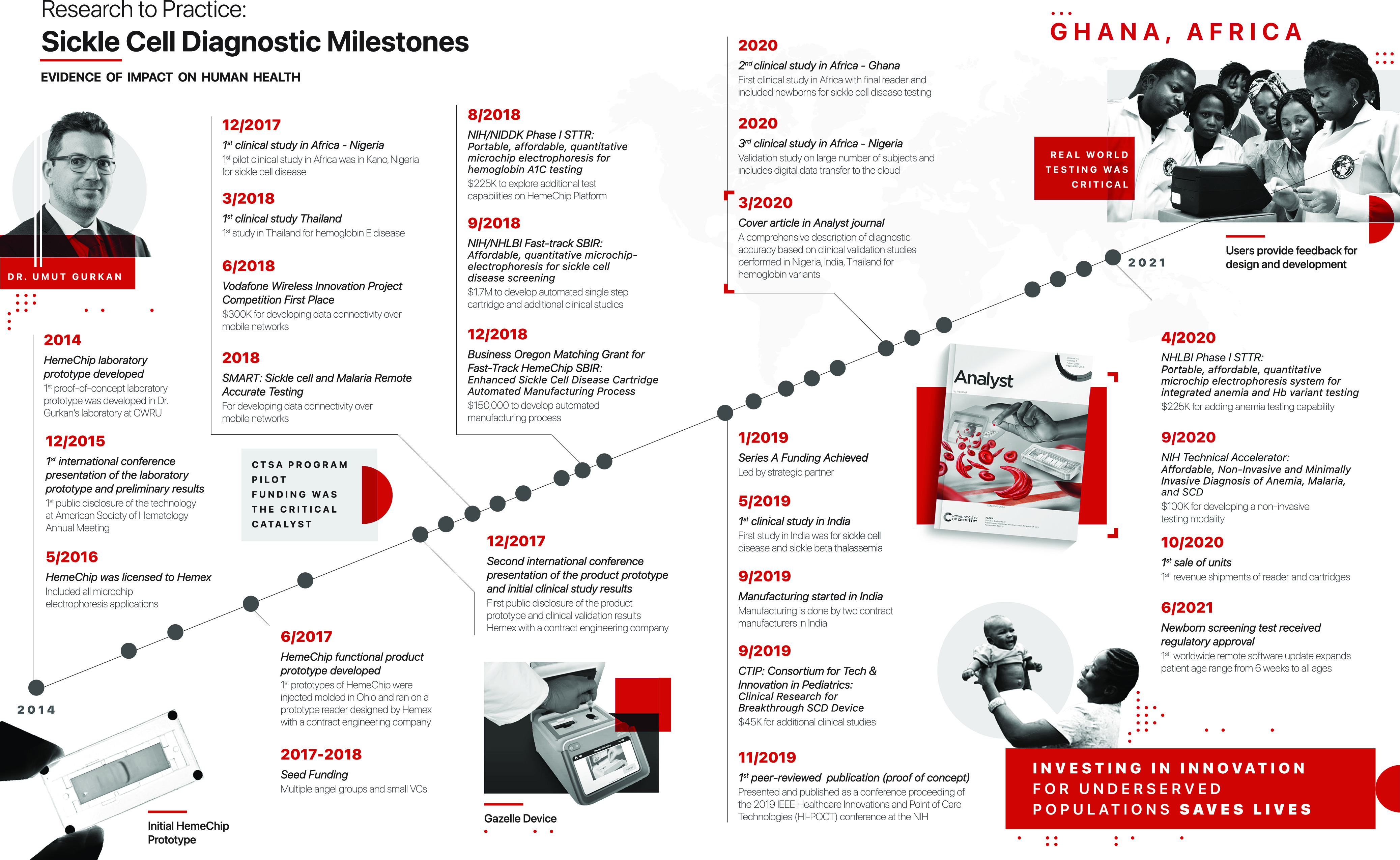
Timeline of key events in the HemeChip’s translation. Sickle cell disease (SCD); Clinical and Translational Science Award (CTSA); National Institutes of Health (NIH); National institute of Diabetes and Digestive and Kidney Diseases (NIDDK); The National Heart, Lung, and Blood Institute (NIHLBI); Small Business Innovation Research (SBIR); Small Business Technology Transfer (STTR).

The results of our document review and interviews are summarized in Fig. 1, Table 1, and Table 2. We conducted five interviews with three key stakeholders and had additional written correspondence with a fourth stakeholder. Figure 1 depicts an annotated timeline of key events during the HemeChip’s translation. Table 1 provides a list of key achievements (e.g., publications, grants). Table 2 contains illustrative quotations that support the barriers and challenges to translation that were identified.

**Table 1.** Key publications, grants, and patents related to the HemeChip in chronological order

Title	Type	Date
Towards a Simple and Reliable Way to Monitor Sickle Cell Disease	Grant	September 1, 2013
Hemoglobin Electrophoresis Biochip for Newborns	Grant	April 1, 2014
Heterogeneous red blood cell adhesion and deformability in sickle cell disease	Publication	November 24, 2014
HemeChip for Point-of-Care Diagnosis of Sickle Cell Disease and Other Hemoglobin Disorders – exploring high volume and low-cost manufacturing of disposable cartridges via plastic injection molding in Cleveland, Ohio	Grant	March 1, 2016
HemeChip for Point-of-Care Diagnosis of Sickle Cell Disease in Newborns – preliminary clinical validation in the US	Grant	March 1, 2016
HemeChip: Point-of-Care Sickle Cell Disease Diagnosis in Low Resource Settings – real world clinical validation in Africa	Grant	March 1, 2016
Sickle cell disease biochip: a functional red blood cell adhesion assay for monitoring sickle cell disease	Publication	March 19, 2016
Emerging point-of-care technologies for sickle cell disease screening and monitoring	Publication	December 1, 2016
Mobile Device Support for Sickle Cell Disease Care in Nigeria	Grant	June 1, 2017
Application of the HemeChip Point-of-Care Device for Real-time Monitoring of Hemoglobin S Levels in Chronically Transfused Patients with Sickle Cell Disease	Grant	November 1, 2017
HemeChip: An Automated Portable Microchip Electrophoresis Platform for Point-of-Care Sickle Cell Disease Screening	Publication	December 7, 2017
Diagnostic systems and methods (US patent #10,768,166)	Patent	March 8, 2018
Diagnostic systems and methods (US patent #10,349,589)	Patent	March 8, 2018
SMART – Sickle and Malaria Accurate Remote Testing	Grant	June 1, 2018
Sickle Cell Disease Biochip Blood Cell Adhesion Test for Emerging Anti-Adhesive Therapies	Grant	September 1, 2018
Diagnostic systems and methods (US patent #10,375,909)	Patent	February 21, 2019
Affordable, quantitative microchip-electrophoresis for sickle cell disease screening	Grant	April 1, 2019
Paper-based microchip electrophoresis for point-of-care hemoglobin testing	Publication	March 2, 2020

**Table 2.** Illustrative quotations of barriers and facilitators to translation

Theme		Illustrative quotation
Barriers to translation	University-industry relations	“The main challenge was in this transitional phase, technology, trying to leave the university and become a part of the company. When it becomes a licensed technology as part of a company, it is very difficult to manage. It’s like the relationship between a lab and a pharma company, for example.” (Gurkan)
Facilitators to translation	CTSA program pilot funding	“So the reason why we need CTSA* grants or Coulter type of grants is especially, even if they are small in size, they help us move the project forward … by allowing us time to collect the critical information, clinical data, and so on. If I had submitted the same project to regular grant opportunities such as an NIH or NSF, they wouldn’t fund those projects, thinking that there’s not enough novelty here or it’s not exciting.” (Gurkan)
		“Once I got the CTSA* first, funding the rest was one thing leading to another, it was just like a domino effect. If the technology and the unmet need are true, then there is that natural momentum that basically makes things happen, but you need that tipping point.” (Gurkan)
	Due diligence	“[We] look at published papers, but not many people look at published patents and expired patents. We do that all the time. So before we do an Invention Disclosure, before we claim that we have an invention, we make sure that we look at the published patents and expired patents and see how they are different and how we can basically prepare ourselves for a future…” (Gurkan)
		“These are really nontraditional, nonacademic research types of things that we had to really report on very carefully. At some point, it was getting really tiresome because nobody asked any questions like this, none of the reviewers, none of the people that [we] presented the technology to. They never asked those types of detailed questions.” (Gurkan)
		“[Dr. Gurkan] attracted us because [he] knew the needs, had a working prototype, credible references and [he]had clinical data. We saw at least 100 technologies and most of them had none of this. [Dr. Gurkan] went deep enough to be credible versus just an idea with no patient data and no market understanding.” (White).
	Collaborations – relationships and synergies	“[Dr. Gurkan was] an excellent collaborator from the beginning. We were able to work together with using the best of both teams. Also, it was very important that we had a shared goal. We all wanted to impact the market and change the lives of the patients who needed it most. That shared goal impacted the product design, cost structure and [the] decision to [do] manufacturing in India.” (White)
		“I consider us friends as well as collaborators. Together we invent things that neither of us could have by ourselves. He can invent things by himself. I can make things by myself, but together, we’re much smarter. That’s how you’ve got to do it.” (Galen)

* Clinical and Translational Science Award (CTSA).

### Development of the HemeChip

The HemeChip was initially conceptualized by a biomedical engineer at CWRU, Dr Umut Gurkan, who spent substantial time researching the unmet needs surrounding SCD. Gurkan’s team developed the prototype for the HemeChip in his laboratory starting in 2013. He used start-up funding provided to him by his academic institution and worked with graduate students who were employed in his laboratory. He received feedback from a SCD expert, Dr Isaac Odame, the Medical Director of the Global Sickle Cell Disease Network at the Center for Global Child Health at The Hospital for Sick Children, Toronto, with whom he developed a subsequent partnership. Dr Odame is a global leader in sickle cell disease research and was an unpaid advisor for this research.

The team at CWRU began applying for local pilot awards through the CTSA pilot funding program and Coulter program in 2014. By the end of 2015, Gurkan presented a protype internationally. After being awarded several pilot grants from both regional entities and foundations, the research team was able to secure larger amounts of funding beginning in 2016. The research team began gaining national recognition around the same time, with Gurkan earning a 2016 National Science Foundation (NSF) Career Award. The HemeChip was officially licensed to Hemex Health in 2016.

Between 2016 and 2019, the HemeChip achieved seed-funding (2017) and Series A funding (2019). During the first five years (2015–2019), the HemeChip received $2.4 million in major federal and/or national grants. The first clinical study also took place during these first five years. In 2020, the first peer-reviewed manuscript on the HemeChip was published [6] and the first patent obtained. Subsequent clinical studies were conducted in India and Africa. In 2020, 5-to-6 years after the initial prototype development, the HemeChip went to market. Over $300,000 in additional funding was awarded in 2020 by the National Institutes of Health (NIH).

Thus far, three patents have been issued and cover several aspects of intellectual property and elements of the commercialization process, with the first patent being issued three years after the first prototype. In mid-2020, a landmark publication outlined the specificity and the diagnostic power of the HemeChip and the results of the international trial, seven years after the first grant funding.

### Barriers to Translation

#### Perceived lack of novelty

The technology behind the HemeChip, electrophoresis, was invented in the 1930s. It is an established standard laboratory technology for separating DNA, RNA, and other molecules using electric current. Gurkan noted that it was challenging to publish work that was based on such a well-known existing technology. The CWRU team spent 2 years trying to publish (Table 1). Reviewers were highly critical of repurposing this technology and pushed Gurkan and his team to conduct large scale international trials to prove the reliability and specificity of HemeChip’s diagnostic capabilities. Despite the perceived lack of novelty associated with repurposing existing technology, this allowed Gurkan to avoid lengthy regulatory applications and approvals. The FDA approved electrophoresis as the standard way to diagnosis Hb variants using a predicate application under the 1976 Medical Device Amendments that established the FDA’s regulation of medical devices and tests. Hemoglobin electrophoresis is included in the WHO’s Essential Diagnostics List for diagnosing sickle cell disease and sickle cell trait [26].

#### High manufacturing costs

The HemeChip encountered challenges with local manufacturing costs. Gurkan had planned to keep manufacturing local, but the high cost of labor and materials in the USA jeopardized the overall affordability of the device. The teams traveled to India where they developed partnerships with manufacturers. During this process, the teams learned that manufacturing the HemeChip in the country where it would be used had unanticipated advantages. One advantage was a greater understanding of who would be using the device, which allowed them to identify practical considerations such as the best way to power and charge the device. However, manufacturing overseas was not without geographic challenges including long distance, international travel, and language barriers.

#### Limited fundraising opportunities

A second financial barrier for the HemeChip was fundraising. Gurkan and Hemex Health relied heavily on a network of connections and the marketing leader of the technology’s licensor company to help raise seed funding for the device. Hemex Health’s experience in commercialization was a large contributor to the fundraising efforts. As the device progressed into the venture capital process, location became a challenge. The HemeChip was developed and located in the Midwest, not near a tech-hub, such as Silicon Valley. There is a smaller pool of venture capital funding available outside of technology-focused cities.

#### Complex university-industry relations

Most clinical and translational researchers are not well versed in the issues relating to Intellectual Property (IP). For example, the process of obtaining and determining ownership of IP, the timing of obtaining IP relative to scientific publication, licensing IP to an external company, and considerations for future development and innovations that use existing IP along with issues of ownership of the original IP. Creation, management, and ownership of IP is an area that universities, who intend to foster entrepreneurship and innovation, need to address in the faculty development of clinical and translational scientists and engineers. Researchers need to partner with outside companies (or create their own company) to get their device manufactured and marketed. However, companies have a vested interest in protecting the IP (i.e., with provisional patents) before any scientific publication can happen. As a result, sometimes the timing is not optimal for the researcher to disseminate their findings. This can be deleterious for the researcher trying to obtain additional research funding, since they have to balance the academic institutional requirements of dissemination of research.

### Facilitators of Translation

#### Obtaining CTSA pilot funding

The CWRU Clinical and Translational Science Award (CTSA) hub awarded a pilot grant toward the HemeChip’s development in 2014. The HemeChip was not eligible for larger federal funding because it focused on device development and commercialization rather than some aspect of basic research or a clinical mechanism. CTSA hubs across the nation must consider their role in awarding funding for unique projects such as the HemeChip. Gurkan stated that he would not have been able to create and disseminate this successful project without opportunities for funding from sources such as the CTSA pilot awards.

#### Support from tech transfer

The university has a Technology (Tech) Transfer Office, which was a key resource in the HemeChip’s translation. Dr Steve Fening, Director of the Case-Coulter Translational Research Partnership, noted that the Tech Transfer Office helped the CWRU team identify funding opportunities. Fening noted that “having multiple small sources of pilot funding from these entities and the CTSA allowed the research team to collect critical information.” The Tech Transfer Office also provided contract and patent services, such as market research and patent law. The CWRU team was able to work with a knowledgeable patent attorney to file patent applications on several points of IP.

#### Partner foundation’s entrepreneurial support

A unique resource located at CWRU is the Case Coulter Translational Research Partnership (CCTRP), which was a joint endowment between the Coulter Foundation and CWRU. CCTRP is focused on entrepreneurial training of investigators. The Coulter Foundation has seven endowed programs across the country. In this case, both a CTSA hub and a Coulter Program exist at the same university and work together to facilitate translation. The two programs are part of a unique infrastructure that provides training and services, like project management, to pilot awardees. The research team was able to receive both entrepreneurial training through the Coulter program as well as consultants for market size calculations. The networks of the CTSA and the Coulter Program also fostered relationships with funders and initially with finding a licensor.

#### Do your due diligence

The HemeChip team identified important prospective factors, such as prior research on the unmet need, IP, patents, and potential partnerships, that influence translation. A critical step in the development of the HemeChip was “to really carefully focus on the unmet need before even getting any funding. The unmet need should drive the invention, not the other way around” (Gurkan). This required extensive research, and when dealing with devices and technology, an element of understanding IP and patents. The CWRU team spent a considerable amount of time early in the HemeChip’s development searching patent libraries. This step in the process of technology and device development is so critical that faculty from the HemeChip research team now require trainees to conduct patent searches during their graduate coursework.

It was also noted that investing time into researching licensure companies was a worthwhile and necessary step in facilitating translation. Due diligence was required between the CWRU team and Hemex Health, studying everything from budgets and spending to manufacturing costs and scalability. The CWRU team had not been previously exposed to this level of questioning. Understanding the processes and practices, like careful documentation, facilitated the HemeChip team in establishing a positive first impression with Hemex Health. When asked about their initial work and impressions of the research, Patti White, CEO of Hemex Health, regarded the depth of research and information gathering as critical.

#### Collaborations: relationships and synergies

Positive relationships between the inventor and the licensor were established early in the development of the technology and continued throughout its development. The HemeChip received pilot funding from two local grant mechanisms with guidance from the Tech Transfer Office. These programs introduced the research team to persons and entities in the local area that were associated with commercialization. This relationship fostered an understanding of the process of translating a device by preparing the research team for commercialization. Second, a strong relationship between Gurkan and Hemex Health created a fruitful and continuous partnership. White noted that the successful relationship and product were due to their shared goal.

Another representative of Hemex Health stated that a strong relationship is needed between both parties to achieve the ultimate outcome of impacting patient care: “He [the inventor] cannot change the world by himself. He needs partners like us to get it out there to actually impact patients. I think that we’re going to make the world better together” (Galen). After the mutual due diligence process, Gurkan and Hemex Health established a highly collaborative relationship that continues to the present.

### Current Status of Impact, Dissemination, and Implementation

In 2020, the researchers associated with HemeChip published results of an international feasibility study with subjects from the USA, India, Africa, and Asia. Clinical sites tested 768 subjects with an overall diagnostic accuracy of 98.4% [6]. HemeChip correctly identified all subjects with hemoglobin S, C, and E variants with 100% sensitivity which is comparable to the reference standard methods. Studies were conducted in settings where the burden of SCD is known to be among the highest in the world including Bangkok, Thailand, Chhattisgarh, India, and Kano, Nigeria. In addition, the feasibility of the administration, training, and use of the HemeChip indicated that local healthcare workers were able to administer the test and analyze samples.

Additional evidence of dissemination and implementation can be found in citations of key publications, grants, or patents in clinical documents. The citation of a key HemeChip publication from 2016 was recently found in a 2019 SCD clinical trial titled “Point-of-care screening for sickle cell disease in low-resource settings: A multi-center evaluation of HemoTypeSC, a novel rapid test” [35]. The website eHealth Africa also lists HemeChip as a device used in the eHealth Africa Laboratory and Diagnostic Facilities [36]. An unanticipated impact of the HemeChip is the economic impact of SCD testing in Africa. The Gates Foundation recently supported an economic impact study that evaluated the benefits of using a low-cost POC diagnostic tool as a screening tool before using a more costly diagnostic apparatus. That study reported that this technique of low-cost rapid screening followed by powerful diagnostics was quicker and more accurate [6].

As the HemeChip continues to be integrated into clinical settings, it is worth noting that the technology and teams behind the HemeChip continue to innovate. The HemeChip’s electrophoresis technology was created to be one component of a larger generalized platform – the Gazelle produced by Hemex Health (Portland, Oregon). Currently, the Gazelle is being developed for other POC diagnostic tests. The team is conducting studies to assess whether the Gazelle Hb Variant could be used to diagnose a wide variety of hemoglobin disorders, such as thalassemias, anemia, as well as other blood diseases, diabetes, or infectious diseases, antimicrobial-resistant microorganisms, and COVID-19.

When reflecting on the process of translation, one person stated, “We have to start with one thing. If you try to do too many things, you do nothing. Do one thing first. But once you have it working, then the other ones are easier” (Galen). With the invention and efficacy of an affordable diagnostic tool on the market, the research team has set their sights on another significant problem associated with SCD, namely therapies. The team has been invited to be a part of the Novartis Biome to further develop their technology alongside therapeutic treatments [37]. The research team will study emerging Gene Therapies with a multi-million grant from the National Heart, Lung, and Blood Institute (NHLBI).

In considering the future use of this device on a global scale, we must address efforts to reduce import and export taxes for low-cost POC diagnostic devices. As of the fall of 2021, the Gazelle Hb Variant has received regulatory approvals and is currently distributed and commercially available in nine countries, including India, Ghana, Nigeria, and other African countries. Obtaining regulatory approvals in nine countries is no simple feat. However, having a singular location for manufacturing (India) presents challenges for keeping the cost of the device at the price point intended, as distributors and government agencies place hefty taxes on imported goods. This illustrates that delivering affordable diagnostic devices to underserved populations is very challenging. Governments could consider reducing or waiving the import duties and taxes for life-saving medical technologies. Investing in innovations for underserved populations, such as the Gazelle Hb Variant, can save lives. There is no current effective method to negotiate lower taxes for devices used specifically for underserved populations.

## Conclusion

This translational science case study was developed from interviews of individuals involved with the research as well as background research on the science. The format for reporting this case study was based on a protocol for conducting case studies of successful translational science [1]. The interviewees may have subjective viewpoints, and so an attempt was made to interview multiple individuals to arrive at a more accurate understanding of the case. Independent corroboration was also sought via document analysis, including publications, grants, and patents. The final determination of both challenges and facilitators that contributed to successful translation came down to evaluation and careful analysis of all the information. Thus, our presentation of this translational science case study may still have gaps or omissions but has been carefully vetted by the researchers involved. The challenges and how they were overcome as well as the key facilitators identified have helped pinpoint areas for consideration in future funding mechanisms and the infrastructure required to facilitate successful translation. Hopefully, the barriers and facilitators to successful translation that are reported here will inform funding agencies regarding designing grant mechanisms to invest in future clinical and translational research institutions. Table 3 provides a coding scheme for this translational research case study. It is hoped that such a scheme will enhance retrieval and subsequent cross-case analysis.

**Table 3.** Classification/coding of case study

Variable	Evidence
Type of intervention [38]	(2) Therapeutic intervention (c) Diagnostic device
Disease/disorder/public health area	Inherited blood disorders, sickle cell disease
Populations affected	People of Indian, African or Hispanic descent
Key markers/milestones	Pilot grants, grants, publications, patents, licensing, fund raising
Key themes-facilitators	Pilot funding; technology transfer from university to industry; entrepreneur training; patent searches (due diligence); collaborations and synergies
Key themes-barriers	Manufacturing costs; fundraising-venture capital for devices; academic-industry bureaucracy; perceived lack of novelty; complex university-industry relations regarding intellectual property
Outcomes achieved	Diagnostic sensitivity & specificity, production/sales to end-users, feasibility of use in low resource countries; ease of use by typical healthcare personnel
Translational stages covered by the case* [39]	T1, T2, T3, T4

* NCATS Translational Stages – T0 = Basic Research, T1 = Preclinical Research, T2 = Clinical Research, T3 = Clinical Implementation, and T4 = Public Health.
